# Invasive Weed *Asystasia gangetica* as a Potential Biomonitor and a Phytoremediator of Potentially Toxic Metals: A Case Study in Peninsular Malaysia

**DOI:** 10.3390/ijerph18094682

**Published:** 2021-04-28

**Authors:** Chee Kong Yap, Weiyun Chew, Khalid Awadh Al-Mutairi, Salman Abdo Al-Shami, Rosimah Nulit, Mohd Hafiz Ibrahim, Koe Wei Wong, Alireza Riyahi Bakhtiari, Moslem Sharifinia, Wan Hee Cheng, Hideo Okamura, Mohamad Saupi Ismail, Muhammad Saleem

**Affiliations:** 1 Department of Biology, Faculty of Science, Universiti Putra Malaysia, UPM, Serdang 43400, Malaysia; chewweiyun@gmail.com (W.C.); rosimahn@upm.edu.my (R.N.); mhafiz_ibrahim@upm.edu.my (M.H.I.); wongkoewei@gmail.com (K.W.W.); 2 Department of Biology, Faculty of Science, University of Tabuk, Tabuk 741, Saudi Arabia; kmutairi@ut.edu.sa; 3 Indian River Research and Education Center, IFAS, University of Florida, Fort Pierce, FL 34945, USA; alshami200@gmail.com; 4 Department of Environmental Sciences, Faculty of Natural Resources and Marine Sciences, Tarbiat Modares University, Noor 46417-76489, Iran; riahi@modares.ac.ir; 5 Shrimp Research Center, Iranian Fisheries Science Research Institute, Agricultural Research, Education and Extension Organization (AREEO), Bushehr 75169-89177, Iran; moslem.sharifinia@yahoo.com; 6 Faculty of Health and Life Sciences, Inti International University, Persiaran Perdana BBN, Nilai 71800, Malaysia; wanhee.cheng@newinti.edu.my; 7 Graduate School of Maritime Sciences, Faculty of Maritime Sciences, Kobe University, Kobe 658-0022, Japan; okamurah@maritime.kobe-u.ac.jp; 8 Fisheries Research Institute, Pulau Pinang 11960, Malaysia; saupi@rocketmail.com; 9 Department of Chemistry, Government Post Graduate College Mirpur, Affiliated Mirpur University of Science and Technology, Mirpur 10250, Pakistan; msaleemqau@yahoo.com

**Keywords:** potentially toxic metals, invasive weeds, phytoremediation, biomonitoring

## Abstract

The invasive weed *Asystasia gangetica* was investigated for its potential as a biomonitor and as a phytoremediator of potentially toxic metals (PTMs) (Cd, Cu, Ni, Pb, and Zn) in Peninsular Malaysia owing to its ecological resistance towards unfavourable environments. The biomonitoring potential of PTMs was determined based on the correlation analysis of the metals in the different parts of the plant (leaves, stems, and roots) and its habitat topsoils. In the roots, the concentrations (mg/kg dry weight) of Cd, Cu, Ni, Pb, and Zn ranged from 0.03 to 2.18, 9.22 to 139, 0.63 to 5.47, 2.43 to 10.5, and 50.7 to 300, respectively. In the leaves, the concentrations (mg/kg dry weight) of Cd, Cu, Ni, Pb, and Zn ranged from 0.03 to 1.16, 7.94 to 20.2, 0.03 to 6.13, 2.10 to 21.8, and 18.8 to 160, respectively. In the stems, the concentrations (mg/kg dry weight) of Cd, Cu, Ni, Pb, and Zn ranged from 0.03 to 1.25, 5.57 to 11.8, 0.23 to 3.69, 0.01 to 7.79, and 26.4 to 246, respectively. On the other hand, the phytoremediation potential of the five metals was estimated based on the bioconcentration factor (BCF) and the translocation factor (TF) values. Correlation analysis revealed that the roots and stems could be used as biomonitors of Cu, the stems as biomonitors of Ni, the roots and leaves as biomonitors of Pb, and all three parts of the plant as biomonitors of Zn. According to the BCF values, in the topsoil, the “easily, freely, leachable, or exchangeable” geochemical fractions of the five metals could be more easily transferred to the roots, leaves, and stems when compared with total concentrations. Based on the TF values of Cd, Ni, and Pb, the metal transfer to the stems (or leaves) from the roots was efficient (>1.0) at most sampling sites. The results of BCF and TF showed that *A. gangetica* was a good phytoextractor for Cd and Ni, and a good phytostabilizer for Cu, Pb, and Zn. Therefore, *A. gangetica* is a good candidate as a biomonitor and a phytoremediator of Ni, Pb, and Zn for sustainable contaminant remediation subject to suitable field management strategies.

## 1. Introduction

The natural ability to withstand harsh environments, such as degraded land, of the invasive weed *Asystasia gangetica* (L.) makes this species a potential biomonitor and phytoremediator for potentially toxic metal (PTM) pollution [1]. Its potential in the biomonitoring and phytoremediation of PTMs is owing to its ecological characteristics, as shown below:

(a) It has a wide geographical distribution. It is a herb originally from India and Sri Lanka [2], but it is now a highly invasive weed species that is widely naturalised in Southeast Asia (Malaysia and Indonesia in particular) and in the Pacific Islands [3], Taiwan [4], and Northern Australia [5,6].
(b) It has high abundance. It produces seeds in large quantities [7]. This causes the weed to be utilised as a cover crop in oil palm plantations [8,9]. It is an evergreen herb that forms mat-like structures that smother more desirable ground plants, hence affecting the biodiversity and agricultural aspects of a particular environment [6].
(c) It is easy to grow and at a fast growth rate [10]. Under experimental polybags in greenhouse conditions, Kumalasari et al. [11] reported that the dry matter yields of both the leaf and stem of *A. gangetica* increased (*p* < 0.001) progressively with age and reached 11.6 g leaf dry matter, 19.0 g stem dry matter/plant, and 30.6 g total dry matter/plant at 90 days after transplanting. Even though the biomass is not substantial when compared with other plants, the fast growth rate of the weeds can still justify *A. gangetica* as a phytoremediator of PTMs.
(d) It is adaptable to different environmental conditions [12], being high shade tolerant [10], it can even grow well under 90% shade [7]. It thrives best in full light and open areas [3,13]. It has high resistance towards metals stress and toxic effects, able to translocate metals from root to shoot, highly resistant towards pathogens and pests, easy adaptability to the climatic conditions of the growth area, and is not part of the food chain as it is not edible by nature [14,15,16,17].
(e) It grows well in various types of soil all year round [18] and it can tolerate high levels of pollutants [19]. Thus, the plant is widely distributed and is found in abundance in unattended open areas such as roadsides and riverbanks [2].
(f) It had been reported to enhance the contents of N, P, and K in the soil and to create a nutrient balance [20]. In addition to contributing N, P, and K to the soil [20], it is also rapidly decomposed [21], and hence can serve as a soil carbon stock [20].
(g) It was proposed as a biomonitor for PTM metal pollution such as Hg [22,23]. In Peninsular Malaysia, it is an invasive non-native weed that can be potentially used as a good biomonitor in different land uses because this weed species is widely distributed here [24].

To study the plant’s potential for phytoremediation (phytoextraction and phytostabilisation) quantitatively, the bioconcentration factor (BCF) and the translocation factor (TF) can be used [25,26,27,28]. For the selection of good hyperaccumulators for the phytoextraction of metals, both BCF and TF are equally crucial to select plants that are capable of phytoextracting metals from the surrounding environment [29].

The BCF is the evaluation of the ability of a plant to accumulate metals from its surrounding environment (such as habitat soil) into its tissues (such as roots, stems, and leaves) [30]. On the other hand, TF is the evaluation of a plant’s ability to transfer the accumulated metals from its below-ground parts (such as roots) to the plant above-ground parts (shoots such as stems or leaves) via the translocation pathway [31].

Reports using plants as biomonitors of anthropogenic sources of PTM pollution are commonly found in the literature [32,33,34,35,36]. These showed that the metal levels in plant parts (leaves and roots) reflect the ambient air pollutant levels and the anthropogenic sources of pollutants in the habitats. In the study by Divan et al. [37], the wild plant species (*Elephantopus mollis*) appeared to have the highest Cd accumulation, suggesting it as a potential bioindicator for Cd. Yildirim and Sasmaz [38] suggested that different parts of various plants could be used as biomonitors. Furthermore, De Paula et al. [39] proposed that the herb *Struthanthus flexicaulis* be utilised as a biomonitor of anthropogenic PTMs. Petrovic et al. [40] reported that the herbaceous perennial weed species, sun spurge (*Euphorbia helioscopia*), served as a good phytoextractor for Cu because it exhibited elevated levels of Cu accumulation, denoting its potential as a Cu-phytoremediator. They recommended the use of this herb as a seasonal biomonitor to screen the general metal pollution levels in the environment.

As there is no detailed study on *A. gangetica* from biomonitoring and phytoremediation aspects, this study aimed to determine the potentials of *A. gangetica* as (1) a biomonitor and (2) a phytoremediator of the PTMs (Cd, Cu, Ni, Pb, and Zn). The evaluation of its potential as a biomonitor of PTMs was based on the correlation analysis of the metals in the different plant parts (leaves, stems, and roots) and its habitat topsoil. Its potential as a phytoremediator of the five PTMs was evaluated based on the TF and BCF values. The results of this study aim at making a weed whose growth had to be regularly controlled into a useful biomonitor and phytoremediator of PTMs.

## 2. Materials and Methods

### 2.1. Sampling Site Descriptions and Soil Collection

**Figure 1:**
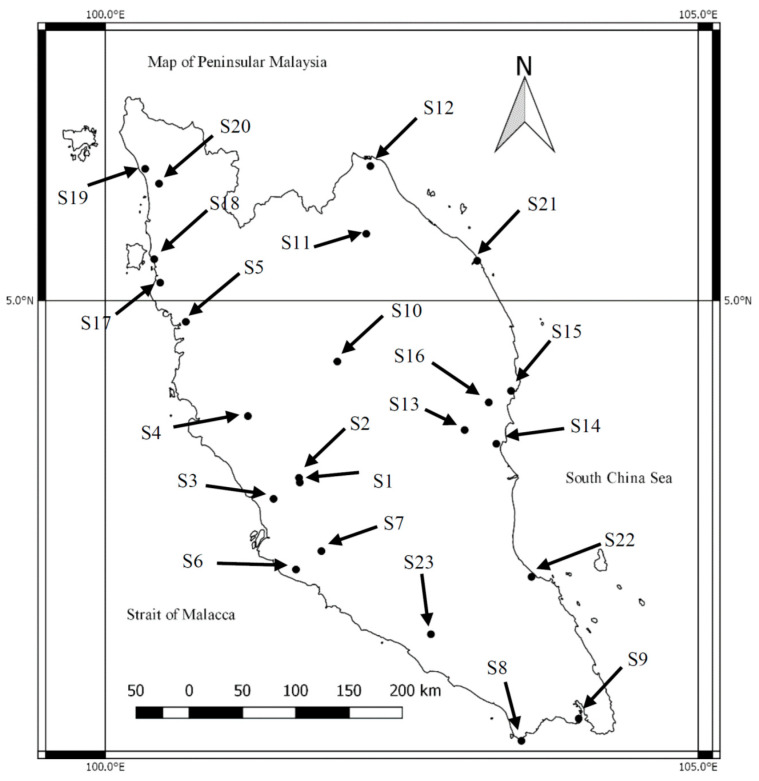
Sampling sites in Peninsular Malaysia (list of sampling sites as in Table 1).

Samplings of topsoils (0–10 cm) and *Asystasia* were done on 23 sites from 8 June 2011 to 17 January 2012, in Peninsular Malaysia (Figure 1; Table 1). Once the samples were collected, they were stored in polyethylene bags.

The sampling sites were selected based on the characteristics of the land uses (Table 1) during the time of sampling. They were categorised as residential area, plantation area, landfill area, rubbish heap, industrial area, and abandoned mining area.

In this study, a sampling site at Juru (S18), which is a known as a polluted active industrialized area in the Juru Industrial Estate [41,42,43,44,45,46], was selected. An abandoned tin mining site at Sg. Lembing (S13) was also selected because it may be polluting the rivers and groundwater with its harmful waste materials such as As, Fe, Cu, Pb, Ni, and Zn [47].

For the plantation area, five sites were sampled, namely, the Kg. Ayer Hitam (S4) site, within the palm oil plantation; the Perah (S10) site, located by a road beside a shop building heavily surrounded by dense trees; the Alor Setar (S19) site, located within a paddy field, close to a greenfield and a road; the Pendang (S20) site, located at the side of a water canal surrounded by paddy field; and the Tg. Gemok (S22) site, located at the side of a farm/orchard close to a housing area.

For the landfill area, four sites were located in close vicinity to the landfill sites. The Matang (S5) open landfill site was located within the landfill facility site (about 300 × 300 m), close to the leachate site about 200 m away [48]. The Sepang (S6) landfill site was located within the landfill facility site (about 400 × 400 m), which was located near the Tanjung Dua Belas Sanitary open landfill in Sepang. The Sg. Kembong (S7) open landfill site was located at the side of a landfill facility (>500 × 500 m) beside a river and it was classified as a type I non-sanitary landfill [49]. The Tanjung Langsat (S9) open landfill site was located at the side of a landfill facility (about 500 × 300 m), which was located in Pasir Gudang industrial area, receiving mainly municipal solid waste [50].

For the rubbish heap area, the three sites (Kuala Krai, S11; Nibong Tebal, S17; Kuala Terengganu, S21) were located with observable municipal waste dumping, legally or illegally. For the residential area, the nine sites were found to have obvious observable residential housing in proximity. These sites were the Kg. Bukit Chandang (S1), the Kg. Bkt. Rasa (S2), the Ijok (S3), the Tanjung Piai (S8), the Kota Bahru (S12), the Kuantan (S14), the Chukai/Kemaman (S15), the Cheneh (S16), and the Pagoh (S23).

The sampling sites, their characteristics, and the parameters of the *A. gangetica* plants sampled from Peninsular Malaysia are presented in Table 1. The number of individuals of *A. gangetica* from each sampling site (2 m^2^) ranged from 7 to 15 individuals. The plant heights from all the sampling sites ranged from 25 to 170 cm. Upon reaching the laboratory, the samples of plants were separated into leaves, roots, and stems. The separated parts of the plants and topsoils were oven-dried at 80 °C for 72 h. The ranges of the water contents in each part of the plant were 73.0–89.1% for leaves, 74.8–89.6% for stems, and 69.9–87.9% for roots.

### 2.2. Metal Analysis

#### 2.2.1. Direct Aqua-Regia Method

The direct aqua-regia method was used to digest the samples of plants and soils. The dried plant samples (leaves, stems, and roots) were digested with 10 mL of nitric acid (HNO_3_; AnalaR grade, BDH 69%) in a digestion tube. For topsoil, the samples were digested with 10 mL of the solvent mixture of nitric acid (HNO_3_; AnalaR grade, BDH 69%) and perchloric acid (HClO_3_; AnalaR grade, BDH 60–70%) in the ratio 4:1. The digestion took place in a digestion block at 40 °C in the first hour and subsequently at 140 °C for 3 h [51]. Whatman No.1 filter paper was used to filter the solution. An acid-washed polyethylene bottle was used to store the solution [51]. The solutions were analysed using a flame atomic absorption spectrometry (FAAS, Perkin Elmer Model AAnalyst 800; Perkin Elmer LLC., Shelton, CT, USA).

#### 2.2.2. Sequential Extraction Technique for Metals

Badri and Aston’s [52] methods of the sequential extraction technique were adopted in this study to achieve the first geochemical fractionations of metals (Cd, Cu, Fe, Ni, Pb, and Zn) from the collected topsoils samples. These were the ‘easily, freely, leachable, or exchangeable’ (EFLE) fractions. Metal fractions were extracted as follows: 10 g of sample was agitated continuously at room temperature for 3 h with 50 mL of 1.0 M ammonium acetate (NH_4_CH_3_COO, pH 7.0).

### 2.3. Quality Control for Metal Analysis

All quality control procedures were conducted, such as the use of acid-washed glassware and equipment and analysis of samples together with procedural blanks and standard solution for each metal, in order to ensure the accuracy of the results. The accuracy of the procedural method for the analysis of Cd, Cu, Fe, Ni, Pb, and Zn was checked with the certified reference material (CRM) (Table 2). The comparisons of the percentage recoveries for the six metals between the certified values of CRM and the measured concentrations are presented in Table 2.

### 2.4. Data Interpretation

#### 2.4.1. Ecological Risk Index (ERI)

The ecological risk index (ERI) was used to determine the potential risk of a single metal in the habitat topsoil [53]. Firstly, the calculation of the contamination factor (Cf) was based on the pollution of a single metal factor as shown in Equation (1).

(1) $$\mathrm{Cf}=\frac{C_{s}}{C_{B}}$$

where C_s_ is the concentration of PTM in topsoil. C_B_ is the background value of each PTM in topsoil. The background concentrations of the metals in the Earth’s upper continental crust (UCC) were taken from Wedepohl [54], which were Cd (0.10 mg/kg), Cu (25.0 mg/kg), Fe (43,000 mg/kg), Ni (56.0 mg/kg), Pb (15.0 mg/kg), and Zn (65.0 mg/kg). Secondly, ERI was calculated based on Equation (2).

(2) $$\mathrm{ERI}=T_{R}\times \mathrm{Cf}$$

where T_R_ is the toxic response factor of a single element. The T_R_ values used in the present study were Cd = 30.0, Cu = 5.00, Ni = 5.00, Pb = 5.00, and Zn = 1.00, according to Hakanson [53]. Based on Hakanson [53], the five classifications for the ERI were “low potential ecological risk” (ERI < 40); “moderate potential ecological risk” (40 ≤ ERI < 80); “considerable potential ecological risk” (80 ≤ ERI < 160); “high potential ecological risk” (160 ≤ ERI < 320), and “very high ecological risk” (ERI ≥ 320).

#### 2.4.2. Calculation of Translocation Factor and Bioconcentration Factor

The translocation factor (TF) and the bioconcentration factor (BCF) were utilised to calculate the plant’s ability to uptake and withstand PTMs. These two indices are commonly used to determine the suitability of plants as good phytoremediators [26,55]. BCF is used to determine the plant’s ability to bioaccumulate PTMs from soils. It is defined as in Equation (3):

(3) $${\mathrm{BCF}}_{\mathrm{root}}=\frac{{\mathrm{Root}}_{\mathrm{metal}}}{{\mathrm{Soil}}_{\mathrm{metal}}}\;{\mathrm{BCF}}_{\mathrm{stem}}=\frac{{\mathrm{Stem}}_{\mathrm{metal}}}{{\mathrm{Soil}}_{\mathrm{metal}}}\;{\mathrm{BCF}}_{\mathrm{leaf}}=\frac{{\mathrm{Leaf}}_{\mathrm{metal}}}{{\mathrm{Soil}}_{\mathrm{metal}}}$$

TF is used to determine the ability of the plant to translocate metals from the roots to the shoots (stem or leaf). It is defined as in Equation (4):

(4) $${\mathrm{TF}}_{\mathrm{stem}}=\frac{{\mathrm{Stem}}_{\mathrm{metal}}}{{\mathrm{Root}}_{\mathrm{metal}}}\;{\mathrm{TF}}_{\mathrm{leaf}}=\frac{{\mathrm{Leaf}}_{\mathrm{metal}}}{{\mathrm{Root}}_{\mathrm{metal}}}$$

The principle of phytoextraction is to remove PTMs from the soil by uptaking and translocating them from the roots of the plant to the leaves and stems (the easily harvested components of the plants).

### 2.5. Data Analysis

All graphical histograms were made by using the KaleidaGraph (Version 3.08, Sygnergy Software, Eden Prairie, MN, USA). In order to reduce the variance [56], Pearson’s correlation analysis was based on log_10_ transformed data of the metals using STATISTICA (Version 10; StatSoft. Inc., Tulsa, OK, USA, 1984–2011). After the log_10_ transformation on the data of Cd, Cu, Ni, Pb, and Zn, the plants and the topsoils showed that all the data were within the normality ranges for skewness (−2 to +2) [57,58,59] and kurtosis (−7 to +7) [57,58].

## 3. Results

### 3.1. Potentially Toxic Metals in Asystasia gangetica

**Figure 2:**
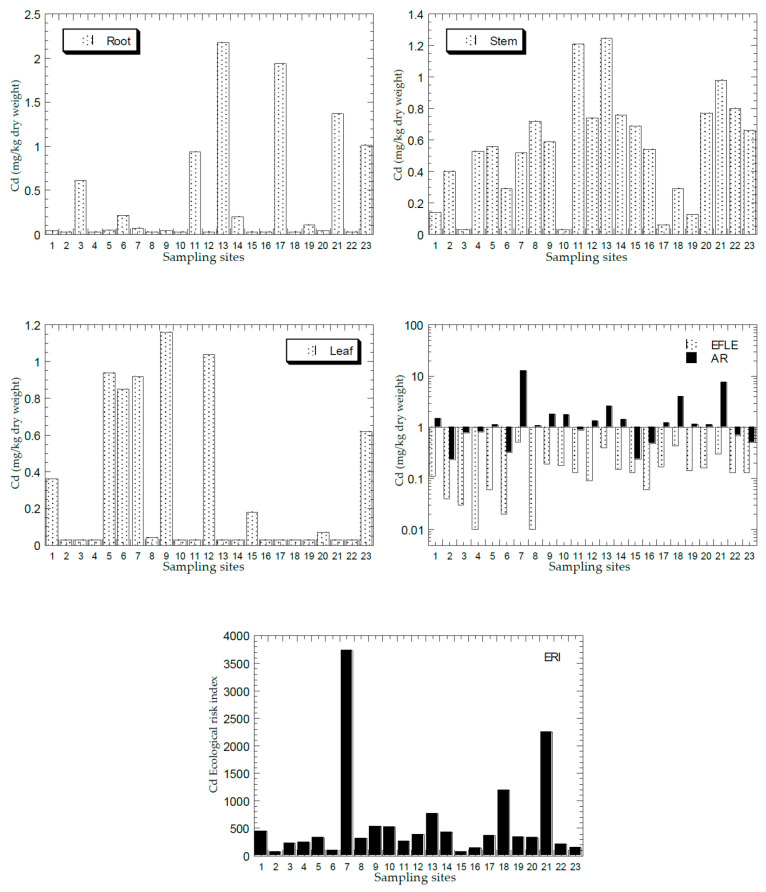
Mean concentrations (mg/kg dry weight) of Cd in the plant parts (Y-axes) and topsoils (total concentration (AR), and geochemical easily, freely, leachable, or exchangeable (EFLE) fractions; ecological risk index (ERI)) in all 23 sampling sites (X-axes). Y-axes for EFLE and AR are drawn based on a logarithmic scale.

**Figure 3:**
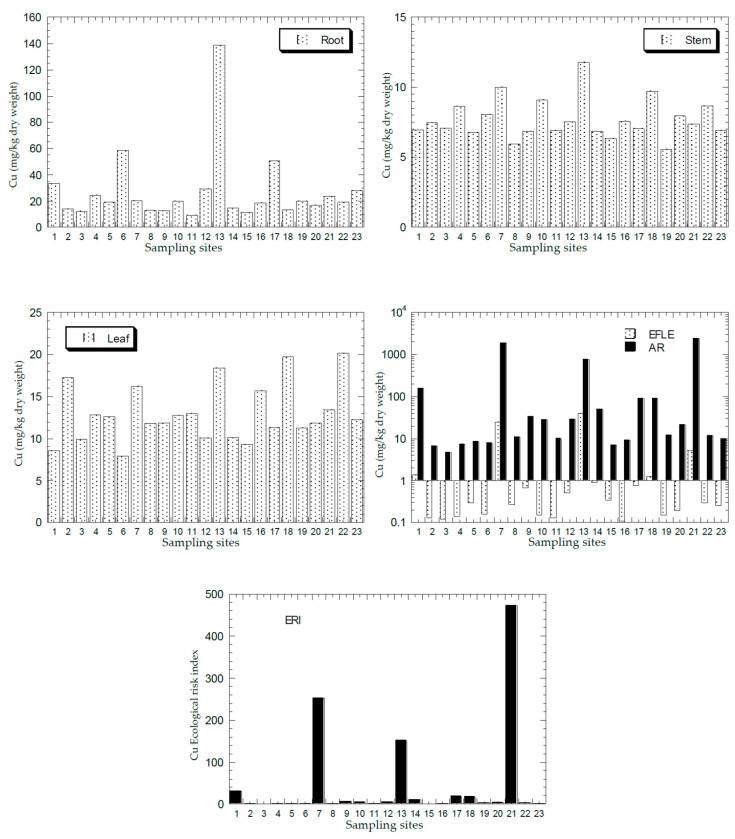
Mean concentrations (mg/kg dry weight) of Cu in the plant parts (Y-axes) and topsoils (total concentration (AR), and geochemical easily, freely, leachable, or exchangeable (EFLE) fractions; ecological risk index (ERI)) in all 23 sampling sites (X-axes). Y-axes for EFLE and AR are drawn based on a logarithmic scale.

**Figure 4:**
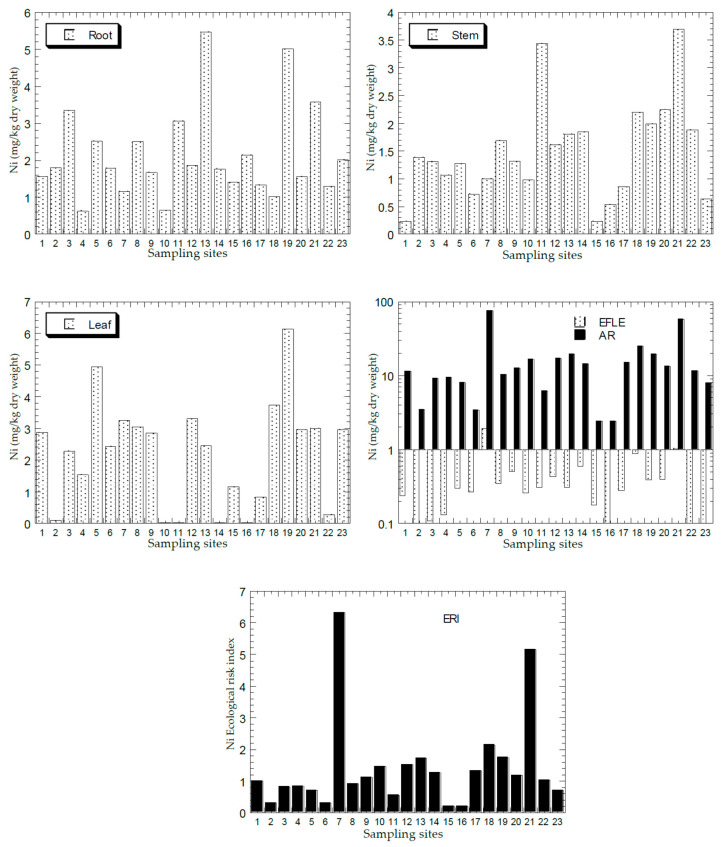
Mean concentrations (mg/kg dry weight) of Ni in the plant parts (Y-axes) and topsoils (total concentration (AR), and geochemical easily, freely, leachable, or exchangeable (EFLE) fractions; ecological risk index (ERI)) in all 23 sampling sites (X-axes). Y-axes for EFLE and AR are drawn based on a logarithmic scale.

**Figure 5:**
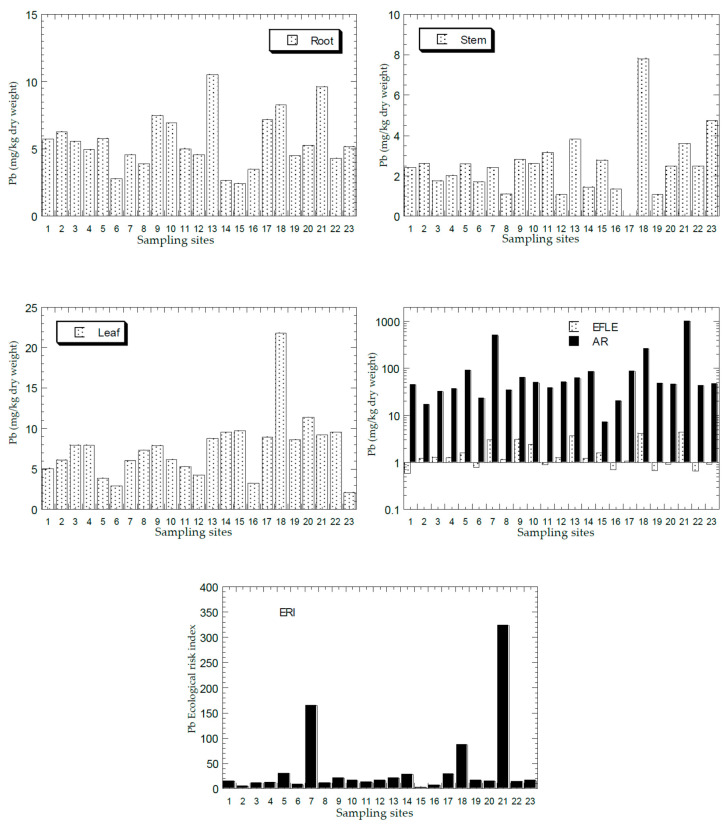
Mean concentrations (mg/kg dry weight) of Pb in the plant parts (Y-axes) and topsoils (total concentration (AR), and geochemical easily, freely, leachable, or exchangeable (EFLE) fractions; ecological risk index (ERI)) in all 23 sampling sites (X-axes). Y-axes for EFLE and AR are drawn based on a logarithmic scale.

**Figure 6:**
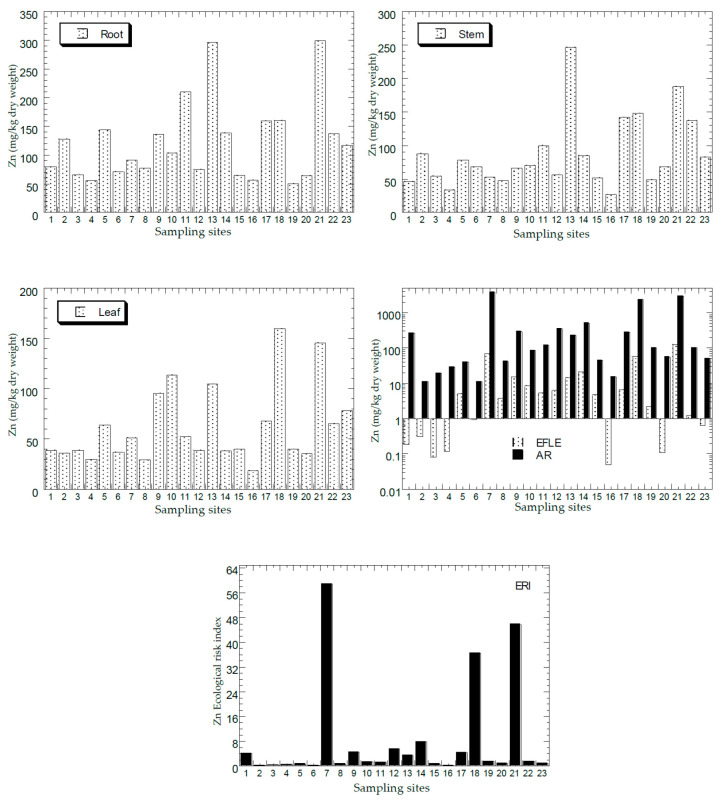
Mean concentrations (mg/kg dry weight) of Zn in the plant parts (Y-axes) and topsoils (total concentration (AR), and geochemical easily, freely, leachable, or exchangeable (EFLE) fractions; ecological risk index (ERI)) in all 23 sampling sites (X-axes). Y-axes for EFLE and AR are drawn based on a logarithmic scale.

Figure 2, Figure 3, Figure 4, Figure 5 and Figure 6 show the mean concentrations of Cd, Cu, Ni, Pb, and Zn in the roots, stems, and leaves of plants from all the sampling sites of this study. The overall statistics of metal concentrations in the roots, stems, and leaves are shown in Table 3.

For the roots (Table 3), the metal concentrations (mg/kg dry weight) for Cd, Cu, Ni, Pb, and Zn ranged from 0.03 to 2.18, 9.22 to 139, 0.63 to 5.47, 2.43 to 10.5, and 50.7 to 300, respectively. For the root samples, S13 (Cd: 2.18) and S17 (Cd: 1.94) were found to have higher Cd concentrations. Among all the sites, S13 had the highest Cu concentration (139) in roots, followed by S6 (58.4). As for Ni, S13 (5.47) and S19 (5.02) had higher concentrations in roots. S9 (7.49), S10 (6.95), S13 (10.5), and S21 (9.62) were found to have higher Pb concentrations. The highest Zn concentrations in roots were found in S13 (296) and S21 (300), followed by S11 (210), S17 (160), and S18 (161). Generally, S13 had higher PTM concentrations in the roots when compared with the other sites, while S21 showed elevated levels of Pb and Zn, and S18 of the level of Zn.

For the stems (Table 3), the metal concentrations (mg/kg dry weight) for Cd, Cu, Ni, Pb, and Zn ranged from 0.03 to 1.25, 5.57 to 11.8, 0.23 to 3.69, 0.01 to 7.79, and 26.4 to 246, respectively. For stems samples, S13 and S11 had higher Cd concentrations (1.25 and 1.21, respectively). S13 was found to have the highest Cu concentration in stems (11.8). It was followed by S7 (10.0) and S18 (5.94). S11 and S21 were found to have higher Ni concentrations in stems (3.69 and 3.44, respectively). The highest concentration of Pb in stems was found at S18 (7.79). As for Zn, S7 (246 µg/g) had the highest concentration, followed by S21 (189). Again, in general, S13 was higher in PTM concentrations in stems when compared with the other sites, while S18 was found to have elevated levels of Cu and Pb, and S21 of the levels of Ni and Zn.

For the leaves (Table 3), metal concentrations (mg/kg dry weight) for Cd, Cu, Ni, Pb, and Zn ranged from 0.03 to 1.16, 7.94 to 20.2, 0.03 to 6.13, 2.10 to 21.8, and 18.8 to 160, respectively. For leaves samples, several sites were found to have the highest concentrations of PTMs. S18 showed the highest levels of Cu, Pb, and Zn in the leaves. Meanwhile, S2, S13, and S21 were found to have elevated levels of Cu. For Ni concentrations, S19 was found to have the highest Ni concentration (6.13), followed by S5 (4.95). The highest Pb concentrations were found at S18 (160), followed by S21 (145) and S10 (114). In general, S18 had the highest concentrations of Cu, Pb, and Zn in leaves, while S13 (an abandoned mining site), S18, and S21 were found to have elevated levels of Pb, and S21 of the level of Cu.

### 3.2. Potentially Toxic Metals in Habitat Topsoils

#### 3.2.1. Total Metal Concentrations and EFLE

The mean concentrations of Cd, Cu, Ni, Pb, and Zn in the topsoil total concentrations (AR) and the EFLE from all the sampling sites are presented in Figure 2, Figure 3, Figure 4, Figure 5 and Figure 6. The overall statistics of the values of topsoil AR and EFLE from the present study are also presented in Table 3. For the topsoil AR, the metal concentrations (mg/kg dry weight) for Cd, Cu, Ni, Pb, and Zn ranged from 0.23 to 12.4, 4.66 to 2363, 2.38 to 75.7, 7.22 to 1004, and 11.0 to 3820, respectively. For the topsoil EFLE, metal concentrations (mg/kg dry weight) for Cd, Cu, Ni, Pb, and Zn ranged from 0.01 to 0.51, 0.11 to 40.1, 0.02 to 1.94, 0.59 to 4.38, and 0.05 to 130, respectively.

Based on Cd AR (Figure 2), four sites (S7, S13, S18, and S21) were found to be significantly higher than the other sites, while the Cd EFLE values of all the sampling sites were below 1.00. Based on Cu AR (Figure 3), four sites (S1, S7, S13, and S21) were found to be significantly higher (>100 mg/kg dry weight) than the other sites, while the Cu EFLE of four sites (S7, S13, and S21) were above 5.0.

Based on Ni AR (Figure 4), two sites (S7, and S21) were found to be significantly higher (>60 mg/kg dry weight) than the other sites, while the Ni EFLE of two sites (S7, and S21) were above 1.0. Based on Pb AR (Figure 5), three sites (S7, S18, and S21) were found to be significantly higher (>200 mg/kg dry weight) than the other sites, while the Pb EFLE of three sites (S13, S18, and S21) were above 3.0. Based on Zn AR (Figure 6), three sites (S7, S18, and S21) were found to be significantly higher (>1000 mg/kg dry weight) than the other sites, while the Zn EFLE of three sites (S7, S18, and S21) were above 40.0. Lum et al. [60] conducted a study in Cameroon in 2011 and reported the PTMs in soils as Cu: 70–179, Pb: 8–130, Zn: 200–971, and Ni: 74–296 mg/kg dry weight.

#### 3.2.2. Ecological Risk Index (ERI)

The mean ERI of Cd, Cu, Ni, Pb, and Zn in the topsoils ERI from all sampling sites is presented in Figure 2, Figure 3, Figure 4, Figure 5 and Figure 6. The overall statistics of the ERI values are also presented in Table 3. The ERI values for Cd, Cu, Ni, Pb, and Zn ranged from 71.2 to 3729, 0.93 to 473, 0.21 to 6.31, 2.41 to 323, and 0.17 to 58.8, respectively.

Three sites (S7, S18, and S21) had significantly higher ERI values for Cd when compared with the other sites (Figure 2). Fourteen sites (S1, S5, S7, S8, S9, S10, S12, S13, S14, S17, S18, S19, S20, and S21) were all categorised as “very high ecological risk” (ERI ≥ 320). Therefore, Cd in the topsoil had a high contribution to the increment of the total ERI values of all metals. Based on Cu ERI (Figure 3), three sites (S7, S13, and S21) were shown to have significantly higher ERI values when compared with the other sites. S7 and S13 were categorised as “high potential ecological risk” (160 ≤ ERI < 320), while S21 as “very high ecological risk” (ERI ≥ 320). Based on Ni ERI (Figure 4), two sites (S7 and S21) were shown to have significantly higher ERI values when compared with the other sites. However, all sites were categorised as “low potential ecological risk” (ERI < 40).

Based on Pb ERI (Figure 5), three sites (S7, S18, and S21) were shown to have significantly higher ERI values when compared with the other sites. Sites S7 and S21 were categorised as “high potential ecological risk” (160 ≤ ERI < 320), while S18 as “considerable potential ecological risk” (80 ≤ ERI < 160). Based on Zn ERI (Figure 6), three sites (S7, S18, and S21) were shown to have significantly higher ERI values when compared with the other sites. However, only S7 and S21 were categorised as “moderate potential ecological risk” (80 ≤ ERI < 160).

S7 consistently showed elevated values of ERI for Cd, Cu, Ni, Pb, and Zn. Hence, S7 was found to be more polluted and had higher ecological risk. The abandoned mining location, S13, was reported to have high ERI for Cd and Cu, because of its waste materials. With elevated levels of PTMs, this area was identified as the source of pollution to rivers and groundwater [47]*.*

S18 at Juru was found to have high ERI for Cd, Pb, and Zn. Juru is known as a polluted active industrial area in the Juru Industrial Estate [41,42,43].

S21, a rubbish heap site, was found to have high ERI for Cd, Cu, Ni, Pb, and Zn. This could be related to municipal wastes including electronic waste from the nearby locations.

### 3.3. Correlations of Potentially Toxic Metals between Topsoil and Plants Parts (Leaves, Stems, and Roots)

The correlation coefficients of Cd, Cu, Ni, Pb, and Zn concentrations between the plant parts (root, stem, and leaf) and the topsoils (AR and EFLE) are presented in Table 4.

For Cd, there were no correlations (or weak and insignificant correlations) found between the three plant parts and the topsoil EFLE (0.04–0.25; *p* < 0.05) and the topsoil AR (0.03–0.17; *p* > 0.05). For Cu, obvious higher positive and significant correlations were found for topsoil EFLE and roots (R = 0.48; *p* < 0.05), stems (R = 0.54; *p* < 0.05), and leaves (R = 0.30; *p* > 0.05) when compared with those of topsoil AR for roots (R = 0.41; *p* > 0.05), stems (R = 0.48; *p* > 0.05), and leaves (R = 0.26; *p* > 0.05).

For Ni, relatively weak R values and insignificant correlations were found between plant parts and topsoil EFLE and AR (R = 0.02–0.40; *p* > 0.05), except for stem and topsoil AR (R = 0.48; *p* < 0.05). For Pb, obvious higher positive and significant correlations were found between root and topsoil AR and EFLE (R = 0.53–0.58; *p* < 0.05), compared with leaf and topsoil EFLE (R = 0.42; *p* > 0.05). For Zn, obvious higher positive significant correlations were found between all the plant parts and topsoils (EFLE and AR (R = 0.44–0.69; *p* < 0.05).

### 3.4. Bioconcentration Factors and Translocation Factors of Potentially Toxic Metals in Asystasia gangetica

**Figure 7:**
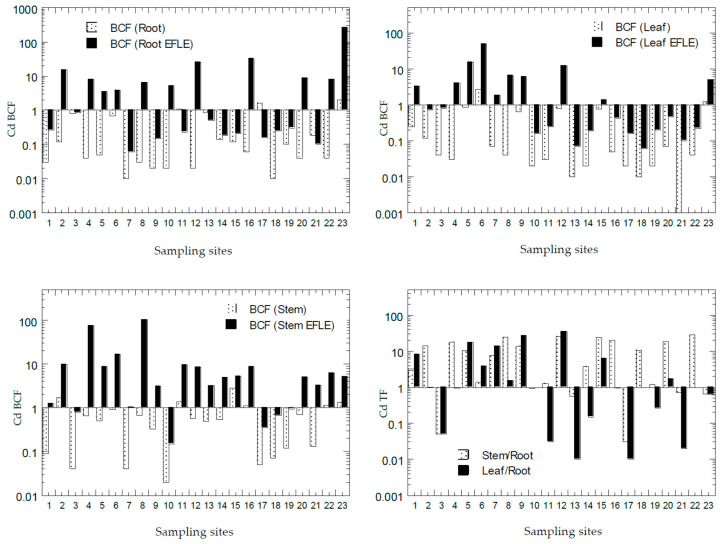
Bioaccumulation factors (BCFs) and translocation factors (TFs) (y-axis) of Cd in all sampling sites (x-axis). Note: BCF (Root) = BCF-1root; BCF (Root EFLE) = BCF-2root; BCF (Leaf) = BCF-1leaf; BCF (Leaf EFLE) = BCF-2leaf; BCF (Stem) = BCF-1stem; BCF (Stem EFLE) = BCF-2stem; TF (Stem/Root) = TF-1; TF (Leaf/Root) = TF-2.

**Figure 8:**
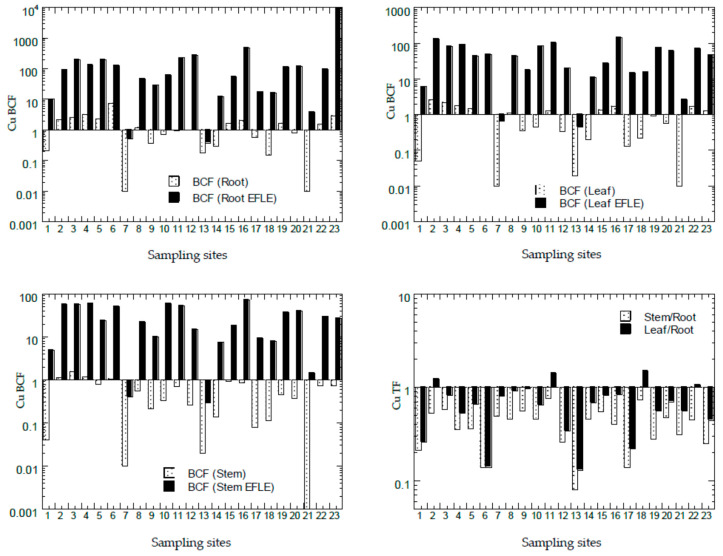
Bioaccumulation factors (BCFs) and translocation factors (TFs) (Y-axes) of Cu in all sampling sites (X-axes). Note: All Y-axes are drawn based on logarithmic scale. BCF (Root) = BCF-1_root_; BCF (Root EFLE) = BCF-2_root_; BCF (Leaf) = BCF-1_leaf_; BCF (Leaf EFLE) = BCF-2_leaf_; BCF (Stem) = BCF-1_stem_; BCF (Stem EFLE) = BCF-2_stem_; TF (Stem/Root) = TF-1; TF (Leaf/Root) = TF-2.

**Figure 9:**
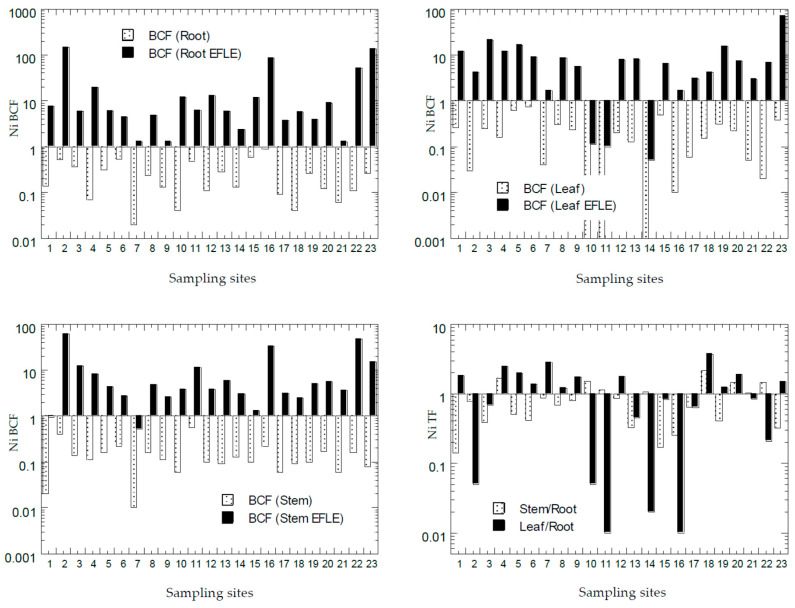
Bioaccumulation factors (BCFs) and translocation factors (TFs) (Y-axes) of Ni in all sampling sites (X-axes). Note: All Y-axes are drawn based on logarithmic scale. BCF (Root) = BCF-1_root_; BCF (Root EFLE) = BCF-2_root_; BCF (Leaf) = BCF-1_leaf_; BCF (Leaf EFLE) = BCF-2_leaf_; BCF (Stem) = BCF-1_stem_; BCF (Stem EFLE) = BCF-2_stem_; TF (Stem/Root) = TF-1; TF (Leaf/Root) = TF-2.

**Figure 10:**
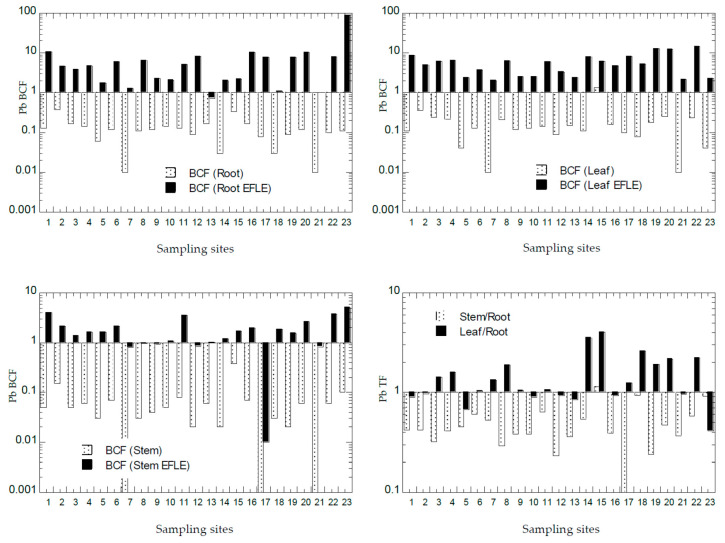
Bioaccumulation factors (BCFs) and translocation factors (TFs) (Y-axes) of Pb in all sampling sites (X-axes). Note: All Y-axes are drawn based on logarithmic scale. N = 23. BCF (Root) = BCF-1_root_; BCF (Root EFLE) = BCF-2_root_; BCF (Leaf) = BCF-1_leaf_; BCF (Leaf EFLE) = BCF-2_leaf_; BCF (Stem) = BCF-1_stem_; BCF (Stem EFLE) = BCF-2_stem_.

**Figure 11:**
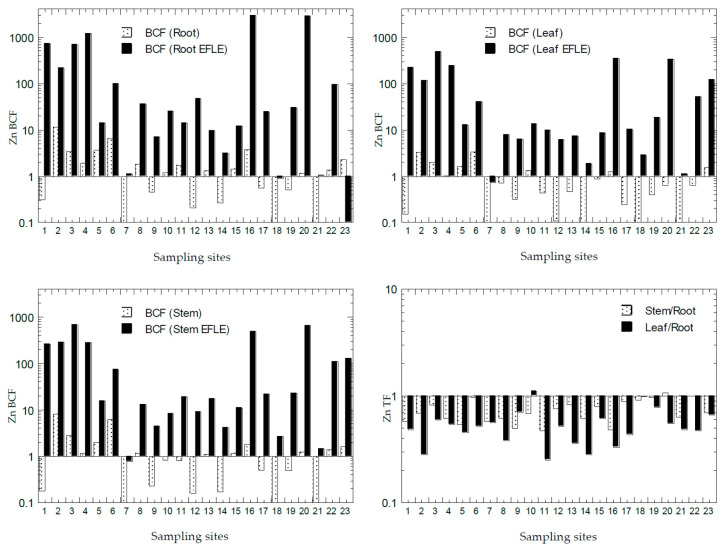
Bioaccumulation factors (BCFs) and translocation factors (TFs) (Y-axes) of Zn in all sampling sites (X-axes). Note: All Y-axes are drawn based on logarithmic scale. BCF (Root) = BCF-1_root_; BCF (Root EFLE) = BCF-2_root_; BCF (Leaf) = BCF-1_leaf_; BCF (Leaf EFLE) = BCF-2_leaf_; BCF (Stem) = BCF-1_stem_; BCF (Stem EFLE) = BCF-2_stem_.

The values of BCF and TF of all PTMs from all the sampling sites from Peninsular Malaysia are presented in Figure 7, Figure 8, Figure 9, Figure 10 and Figure 11. The overall statistics of the values of TF and BCF are also given in Table 3.

The values of BCF-1_root_ for Cd, Cu, Ni, Pb, and Zn ranged from 0.01 to 2.00, 0.01 to 7.52, 0.02 to 0.89, 0.01 to 0.37, and 0.02 to 11.6, respectively. The values of BCF-2_root_ for Cd, Cu, Ni, Pb, and Zn ranged from 0.06 to 258, 0.37 to 9254, 1.27 to 148, 0.73 to 86.1, and 0.00 to 2931, respectively. The values of BCF-1_leaf_ for Cd, Cu, Ni, Pb, and Zn ranged from 0.00 to 2.66, 0.01 to 2.61, 0.01 to 0.72, 0.01 to 1.35, and 0.01 to 3.33, respectively. The values of BCF-2_leaf_ for Cd, Cu, Ni, Pb, and Zn ranged from 0.06 to 48.8, 0.46 to 148, 0.05 to 70.0, 2.00 to 14.6, and 0.74 to 481, respectively. The values of BCF-1_stem_ for Cd, Cu, Ni, Pb, and Zn ranged from 0.02 to 2.89, 0.00 to 1.52, 0.01 to 0.56, 0.00 to 0.38, and 0.01 to 7.99, respectively. The values of BCF-2_stem_ for Cd, Cu, Ni, Pb, and Zn ranged from 0.15 to 105, 0.29 to 72.0, 0.52 to 61.0, 0.01 to 5.10, and 0.76 to 674, respectively. The values of TF-1_stem_ for Cd, Cu, Ni, Pb, and Zn ranged from 0.03 to 27.8, 0.08 to 0.75, 0.14 to 2.18, 0.00 to 1.14, and 0.47 to 1.07, respectively. The values of TF-2_leaf_ for Cd, Cu, Ni, Pb, and Zn ranged from 0.01 to 35.4, 0.13 to 1.50, 0.01 to 3.70, 0.41 to 4.02, and 0.25 to 1.10, respectively.

Based on the values of Cd BCF in the roots (Figure 7), there were only two sites (S17 and S23) with BCF-1_root_ > 1.0, while 10 sites were found with BCF-2_root_ > 1.0. Based on the values of Cd BCF in the leaves (Figure 7), there were only two sites (S6 and S23) with BCF-1_leaf_ > 1.0, while 10 sites were found with BCF-2_leaf_ > 1.0. Based on values of Cd BCF in the stems (Figure 7), there were only six sites (S1, S11, S15, S16, S22, and S23) with BCF-1_stem_ > 1.0, while 18 sites were found with BCF-2_stem_ > 1.0. This showed that the Cd in the topsoil EFLE fraction could be more easily transferred to the roots, leaves, and stems when compared with those in the topsoil total concentrations of Cd. Based on the values of Cd TF (Figure 7), 17 sites (74%) with TF-1_stem_ > 1.0 were found, while 9 sites (39%) were found with TF-2_leaf_ > 1.0. This showed that Cd transfer to the stems from the roots was very efficient in most sampling sites, while Cd transfer to the leaves from the roots was less efficient. Values of BCF > 1.0 and TF > 1.0 make *A. gangetica* a potential phytoextraction agent for Cd [26].

Based on values of Cu BCF in the roots (Figure 8), there were 11 sites with BCF-1_root_ > 1.0, while 21 sites appeared to have BCF-2_root_ > 1.0. Based on the values of Cu BCF in the leaves (Figure 8), there were 10 sites with BCF-1_leaf_ > 1.0, while 21 sites were found with BCF-2_leaf_ > 1.0. Based on values of Cu BCF in the stems (Figure 8), there were only 3 sites (S2, S3, and S4) with BCF-1_stem_ > 1.0, while 21 sites were found with BCF-2_stem_ > 1.0. This showed that the Cu in the topsoil EFLE fraction could be more easily transferred to the roots, leaves, and stems when compared with those in the topsoil total concentrations of Cu. Based on the values of Cu TF (Figure 8), all 23 sites (100%) had TF-1_stem_ < 1.0, while there were 19 sites (83%) were found with TF-2_leaf_ < 1.0. This showed that the transfer of Cu from the roots to the leaves was less efficient and limited, while there was no apparent Cu transfer to the stems from the roots. With most of the values of BCF >1.0 and TF < 1.0, *A. gangetica* has the potential to be used in phytostabilisation of Cu [26].

Based on values of Ni BCF in the roots (Figure 9), all 23 sites had BCF-1_root_ < 1.0, while all 23 sites had BCF-2_root_ > 1.0. Based on the values of Ni BCF in the leaves (Figure 9), all 23 sites had BCF-1_leaf_ < 1.0, while 20 sites had BCF-2_leaf_ > 1.0. Based on the values of Ni BCF in the stems (Figure 9), all 23 sites had BCF-1_stem_ < 1.0, while 21 sites had BCF-2_stem_ > 1.0. This showed that the Ni in the topsoil EFLE fraction could be more easily transferred to the roots, leaves, and stems when compared with those in the topsoil total concentrations of Ni. Based on values of Ni TF (Figure 9), there were 7 sites (30%) with TF-1_stem_ > 1.0, while there were 12 sites (52%) found with TF-2_leaf_ > 1.0. This showed that the transfer of Ni from the roots to the leaves was more efficient than those to the stems. With the values of BCF > 1.0 and TF > 1.0, *A. gangetica* has the potential to be used in phytoextraction of Ni [26].

Based on values of Pb BCF in the roots (Figure 10), all 23 sites were found with BCF-1_root_ < 1.0, while 21 sites were found with BCF-2_root_ > 1.0. Based on values of Pb BCF in the leaves (Figure 10), there was only 1 site (S15) with BCF-1_leaf_ > 1.0, while all 23 sites were found with BCF-2_leaf_ > 1.0. Based on values of Pb BCF in the stems (Figure 10), all 23 sites had BCF-1_stem_ < 1.0, while 17 sites were found with BCF-2_stem_ > 1.0. This showed that the Pb in the topsoil EFLE fraction could be more easily transferred to the roots, leaves, and stems when compared with those in the topsoil total concentrations of Pb. Based on values of Pb TF (Figure 10), 22 sites (96%) were found with TF-1_stem_ < 1.0, while 9 sites (39%) were found with TF-2_leaf_ < 1.0. This showed that Pb transfer to the leaves from the roots was efficient in most sampling sites, while Pb transfer to the stems from the roots was less efficient. With most sites with the values of BCF > 1.0 and TF < 1.0, *A. gangetica* has the potential to be used in the phytostabilisation of Pb [26].

Based on the values of Zn BCF in the roots (Figure 11), there were 14 sites with BCF-1_root_ > 1.0, while 21 sites were found with BCF-2_root_ > 1.0. Based on the values of Zn BCF in the leaves (Figure 11), there were 7 sites with BCF-1_leaf_ > 1.0, while 22 sites had BCF-2_leaf_ > 1.0. Based on values of Zn BCF in the stems (Figure 11), there were 12 sites with BCF-1_stem_ > 1.0, while 22 sites were found with BCF-2_stem_ > 1.0. This showed that the Zn in the topsoil EFLE fraction could be more easily transferred to the roots, leaves, and stems when compared with those in the topsoil total concentrations of Zn. Based on the values of Zn TF (Figure 11), 22 sites (96%) were found with TF-1_stem_ < 1.0 and TF-2_leaf_ < 1.0. This showed that Zn transfer to the leaves and stems from the roots was less efficient and limited in almost all sampling sites. With most sites with the values of BCF >1.0 and TF < 1.0, *A. gangetica* has the potential to be used in phytostabilisation of Zn [26]. Subha and Srinivas [61] reported BCF: Cd (2.68), Cu (1.48), Ni (6.80), Pb (1.02), and Zn (1.27) in the common marsh buckwheat *Polygonum glabrum* collected from The Hussain Sagar Lake, Hyderabad, Telangana, India.

## 4. Discussion

### 4.1. Biomonitoring of Potentially Toxic Metals

In this study, the potential of *Asystasia* being a biomonitor of PTMs was mainly based on (1) accumulation of PTMs and (2) the correlations of PTMs between the topsoil and the plants parts.

Based on Baker and Brooks [62], *A. gangetica* was found to be not a hyperaccumulator weed plant of PTMs because all three parts of the plants (leaves, stems, and roots) from all the sites did not accumulate high concentrations (mg/kg) of Cd, Cu, Ni, and Pb from the habitat topsoils. Baker and Brooks [62] stated that hyperaccumulator plants are those that can accumulate high concentration of metals from the soils (>1000 mg/kg of Cu, Ni, or Pb; >10,000 mg/kg of Zn; 100 mg/kg of Cd).

Correlation analysis revealed that the roots and stems could be used as biomonitors of Cu, while stems as biomonitors of Ni, roots and leaves as biomonitors of Pb, and all three plant parts as biomonitors of Zn. The higher correlations (R values) between plant and topsoil EFLE than between plant and topsoil total metal concentrations could be because of the metals in the EFLE fraction being more easily assimilated and accumulated in the plant parts [63].

These positive and significant correlations indicated the plant parts of *Asystasia* were able to reflect the metal concentrations (especially of the EFLE fraction) in the habitat topsoils. The positive relationships of metals between the topsoil EFLE fractions and plant roots indicated a close relationship between soil metal concentration and root metabolism, which should be further examined to understand how soil metal concentrations could affect the root metal accumulation efficiency. According to Gallagher et al. [64], when the soil metal concentration exceeded the plant tolerance limit, growth and metabolism would be inhibited and, eventually, the plant species would be excluded from the site vegetation assemblage even though there were still seeds present in the regional pool. Therefore, the roots, leaves, and stems of *Aystasia* are good biomonitors of Cu, Ni, Pb, and Zn pollution in their habitat environmental soils.

The main uptakes of pollutants from the surroundings into the plants are from deposition on leaves and absorptions from root cells [34,36,65] This could be the reason the roots were found to be reflective of Cu, Pb, and Zn in the habitat topsoils. Metals bound in the soils must be mobilised before they can be absorbed by the root cells of the plants [66]. Mobilised metals penetrate the roots either through apoplastic or symplastic pathways [66]. Metals are capable of being transported to the entire plant via the xylem or phloem [67].

### 4.2. Asystasia as Phytoextractor of Cd and Ni

The use of other plants and weeds (both invasive and non-invasive) as phytoextractor of Cd and Ni as reported in the literature (Table 5) supported the present finding using *A. gangetica*. For example, Wei et al. [68] recommended the potential use of the invasive plant species, *Chromolaena odorata*, *Bidens pilosa,* and *Praxelis clematidea*, as candidates for soil Cd phytoextraction based on their abilities to accumulate metals and their advantages in growth and tolerance traits. Favas et al. [69] studied the correlation between Ni concentration in the soil and its concentration in the non-invasive plant (*Alyssum serpyllifolium*) in the ultramafic areas of Portugal. Yu et al. [70] reported that the non-invasive *Celosia argentea* could potentially decontaminate Cd-contaminated soils by acting as a phytoextractor. They revealed positive results on Cd phytoextraction in Cd-contaminated soils using *C. argentea*.

In this study, the results showed that the phytoextraction process helped to concentrate Cd and Ni in the roots and stems. Phytoextraction, the absorption and accumulation of PTMs in the plant shoots, and their removal from the treatment site through harvesting the plant parts is one of the many strategies for the phytoremediation of the soil [26,83]. This method requires the uptake of pollutants from the plant roots and the translocation of the metals to the other parts (stems and leaves) of the weeds [15,84]. This is followed by the biomass harvest of theses plant parts for safe disposal of the accumulated metals.

However, it should be noted that many abiotic factors could influence the efficiency of the phytoextraction processes, such as physico-chemical properties of soil, metal bioavailability to the weeds, metal speciation, climatic conditions, and the weed’s characteristics [14,85,86,87].

In theory, the weeds that act as phytoextractors could accumulate massive amounts of pollutants [85]. However, the suitability of a plant as a phytoextractor species for PTMs is based on the metal accumulation in the shoot (stems and leaves) and the shoot biomass. Being a non-hyperaccumulator, the phytoextraction approach that fits *A. gangetica* well is the relatively higher above ground biomass production owing to its fast growth rate despite its lower metal accumulation. This had also been reported in plants such as *Brassica juncea* [15,88].

Ali et al. [89] stated that the phytoextraction ability of the multi-cut plant species (*Trifolium* spp.) surpassed those of mono-harvest plant species. Weeds are more favourable as compared with shrubs and trees because weeds have literal shorter life cycles, a higher growth rate, higher resistance towards abiotic stresses, and are able to produce more biomass [90]. Hence, in the case of *A. gangetica*, being a non-hyperaccumulator weed of PTMs, the phytoextraction requires several harvesting periods in order to remove PTMs to acceptable levels so as to reduce the potential risk for food chain contamination.

### 4.3. Asystasia as Phytostabiliser of Cu, Pb, and Zn

The use of other plants and weeds (both invasive and non-invasive) as phytostabiliser of Cu, Pb, and Zn as reported in the literature (Table 6) supported the present finding using *A. gangetica*. For example, Drozdova et al. [91] studied the potential of phytoextraction and phytostabilisation of non-invasive *Brassica campestris* for the concentrations of Cd, Cu, Ni, Pb, and Zn in the plant organs (leaves, roots, stems, and inflorescences), and the BCF and TF. Mataruga et al. [92] reported that the BCF and TF factors indicated that the non-invasive elm (*Ulmus glabra*) was suitable for the phytostabilisation of As, Cu, Cr, Ni, and Pb.

Yoon et al. [26] reported the native plants (*Phyla nodiflora)* were suitable phytostabilisers of Cu and Zn, mainly attributed to *P. nodiflora*’s Cu and Zn accumulating abilities in its shoots (TF = 12 and 6.3). They also recommended *Gentiana pennelliana* as a good candidate for the phytostabilisation of Pb, Cu, and Zn (BCF = 11, 22, and 2.6, respectively) in polluted sites. Santos et al. [97] reported that the salt marsh non-invasive *Tamarix africana* helps to stabilise the natural condition of soils, and thus serve as a good phytostabilising agent for saline-contaminated soils. The two main mechanisms of the tolerance in the *T. africana* were its high excretion of the elements through its salt glands and the relatively low translocation of metals from its roots to the other plant parts. Varun et al. [102] reported the weed *Abutilon indicum* displayed BCF >1 at all concentrations. *A. indicum* had translocated most of the metals in its root.

According to Patra et al. [83], the plants phytostabilised the soils by immobilising the pollutants in the rhizospheric region through adsorption or precipitation, thus preventing the pollutants from entering the environment as well as into the food chain of the ecosystem [15,85,103]. Phytostabilisers function through inhibiting the movements of PTMs into the ecosystem, environment, or food chain, via various mechanisms in the roots of the plants such as adsorption, precipitation, and complexation [104]. Phytostabilisation involves metal immobilisation for metal toxicity reduction in the roots, eliminating toxic metal bioavailability in soils. Metals accumulated by *A. gangetica* will be channeled to the root tissues through phytostabilisation processes or transported through xylem vessels to the aerial parts of the plant.

### 4.4. General Discussion

The three sites (S7, S18, and S21) with significant elevated PTM levels in the topsoils showed lower BCF-1 values than the other sites. It was possible that the defensive mechanisms of the plants acted to mitigate the over accumulation of PTMs in highly contaminated topsoils [105]. This behaviour was similar to that reported in a study on *H. verticillate* where the plant gradually decreased the rate of metal uptake with increasing metal toxicity. The plant displayed resistance to metal stress by increasing antioxidant activities to prevent unessential elements (Pb and Cd) from hampering biological metabolism [106]. Moreover, the bioavailability of PTMs in the soil depends on the physico-chemical properties in the soil (pH, organic matter, root exudates, microbial biomass, and competitive cations) [107].

In the literature, many studies documented the use of weeds as phytoremediators of metals [26,27,28,102,108,109]. Generally, the translocations of Cu and Zn from roots to stems and leaves of plants from across the sampling sites were low (TF < 1). Plants from landfill sites (S5, S6, S7, and S9) had high TF-1 and TF-2 values of Cd, Ni, and Pb (TF > 1). This showed the plants were actively translocating toxic metals from the roots. These plants have potentials as phytoextractors with their high BCF values (>1) and as phytostabilisers with their high BCF (>1) and low TF (<1) values [26].

Hence, based on the BCF values of Cd, Cu, Ni, Pb, and Zn, the metals in the topsoil EFLE fraction could be more easily transferred to the roots, leaves, and stems when compared with those in the topsoil total concentrations of the metals. Based on the TF values of Cd, Ni, and Pb, the metal transferred to the stems (or leaves) from the roots was efficient (>1.0) in most sampling sites. Therefore, the results of BCF and TF showed that *A. gangetica* would be a good choice for use as a phytoextractor of Cd and Ni, and as a phytostabiliser of Cu, Pb, and Zn.

*Asystasia* from the industrial site at S18 had lower TF_leaf_ (Cu, Pb, Ni, and Zn) than weeds in the industrial areas of Islamabad [90] and the contaminated urban area of Florida [26]. Most of the plants sampled in the plantation areas had lower TF_leaf_ (Cd, Cu, Ni, Pb, and Zn) than plants from contaminated urban areas in Florida [26] and the industrial areas in Islamabad [90]. Plants sampled in the mining area (S13) had higher TF_leaf_ values than those of plants from the mining area of Mahad AD’Dabah [55]. The plants from the landfill areas had lower TF_leaf_ (Cu, Pb, and Zn) than the plants from the waste dumpsites of South-eastern Nigeria [110]. The rubbish heap plants generally had lower TF_leaf_ (Cu, Pb, and Zn) than the plants from the waste dumpsites in South-eastern Nigeria [110].

The phytoremediation using *Asystasia* is also classified as green technology owing to (i) its environmental friendly approach that maintains and does not destroy the site and (ii) its facilitation of the restoration process after the excavation of the site [83].

## 5. Conclusions

The current study analysed PTM levels in the topsoils and various parts (leaf, stem, and root) of *A. gangetica* plants collected from Peninsular Malaysia. Among all the sites, an abandoned mining site (S13) showed the highest levels of Cd, Cu, Ni, Pb, and Zn in the roots, while a rubbish heap site (S21) was found to have the highest levels of Ni and Zn in stems and roots, respectively, and an industrial site at Juru (S18) showed the highest concentrations of Pb and Zn in the leaves. Correlation analysis revealed that the roots and stems could be used as biomonitors of Cu, while stems as biomonitors of Ni, roots and leaves as biomonitors of Pb, and all the three plant parts as biomonitors of Zn. Based on the BCF values of Cd, Cu, Ni, Pb, and Zn, the metals in the topsoil EFLE fraction could be more easily transferred to the roots, leaves, and stems when compared with those in the topsoil total concentrations of the metals. Hence, we conclude that the results of BCF and TF indicate that *A. gangetica* is a good phytoextraction agent for Cd and Ni, and a good phytostabilisation agent for Cu, Pb, and Zn.

## Figures and Tables

**Table 1:** Sampling sites, their characteristics, and some parameters of the *Asystasia gangetica* plants sampled from Peninsular Malaysia.

No.	Sampling Site	Date/Month/Year	Characteristics	N	PH (cm)	Leaf (WC; %)	Stem (WC; %)	Root (WC; %)
S1	Kg. Bkt. Chandang	8/6/2011	Residential	15	51.1	84.3	78.2	70.3
S2	Kg. Bkt. Rasa	21/6/2011	Residential	14	93.0	84.7	83.5	69.9
S3	Ijok	21/6/2011	Residential	16	59.1	73.0	74.8	28.9
S4	Kg. Ayer Hitam	26/6/2011	Plantation	15	65.3	77.1	85.6	74.4
S5	Matang	27/6/2011	Landfill	15	122.0	81.9	82.5	73.8
S6	Sepang	2/7/2011	Landfill	14	44.4	78.3	84.7	74.9
S7	Sg. Kembung	2/7/2011	Landfill	7	90.7	80.5	84.1	69.7
S8	Tanjung Piai	9/7/2011	Residential	11	97.7	83.6	87.5	80.7
S9	Tanjung Langsat	10/7/2011	Landfill	10	83.0	85.2	81.7	87.9
S10	Perah, Kuala Lipis	15/7/2011	Plantation	13	65.0	84.3	83.1	75.0
S11	Kuala Krai	15/7/2011	Rubbish heap	13	61.2	84.5	84.6	79.0
S12	Kota Bharu	16/7/2011	Residential	11	63.6	88.6	88.5	84.0
S13	Sg. Lembing	22/7/2011	Abandoned mining	13	44.8	74.5	76.2	64.8
S14	Kuantan	22/7/2011	Residential	9	96.1	83.2	84.5	75.5
S15	Chukai/Kemaman	23/7/2011	Residential	13	31.4	83.0	79.2	74.2
S16	Cheneh	23/7/2011	Residential	12	133.0	85.5	79.8	75.1
S17	Nibong Tebal	2/8/2011	Rubbish heap	10	67.5	85.3	84.2	83.3
S18	Juru	2/8/2011	Industrial	11	54.5	82.7	84.2	77.2
S19	Alor Setar	3/8/2011	Plantation	15	47.7	84.7	81.7	73.7
S20	Pendang	3/8/2011	Plantation	7	38.6	84.4	89.6	73.2
S21	Kuala Terengganu	16/11/2011	Rubbish heap	12	83.8	89.1	86.9	86.1
S22	Tg. Gemok	17/11/2011	Plantation	10	107.5	84.4	83.2	66.3
S23	Pagoh	17/1/2012	Residential	12	107.1	80.9	76.7	68.9

Note: WC = water content (%). PH = plant height. N = number of individuals sampled.

**Table 2:** Comparisons of metals analysis recovery percentages with the certified reference materials (CRMs).

CRM	Cd	Cu	Fe	Ni	Pb	Zn
NSC DC73319 Soil China	110.7%	85.0%	NA	NA	99.8%	99.7%
MESS-3 NRC	NA	93.1%	NA	102.0%	115.6%	82.8%
TH-1 sediment Canada	102.4%	92.9%	95.6%	112.3%	100.0%	110.2%
SRM 1547	NA	NA	105.6%	NA	NA	114.9%
IAEA soil-5	156.3%	91.3%	NA	103.0%	115.7%	94.8%

Note: NA—data not available.

**Table 3:** Overall statistics of metal concentrations (mg/kg dry weight) in the plant parts, topsoils (total metal concentration (AR) and geochemical easily, freely, leachable, and exchangeable (EFLE) fractions, and ecological risk index (ERI)), and values and ratios of translocation factor (TF) and bioconcentration factor (BCF) from the present study. N = 23.

		Plant			Topsoils			TF-1	TF-2	BCF-1_root_	BCF-2_root_	BCF-1_leaf_	BCF-2_leaf_	BCF-1_stem_	BCF-2_stem_
Metal		Root	Stem	Leaf	EFLE	AR	ERI	Stem/Root	Leaf/Root	Root/AR	Root/EFLE	Leaf/AR	Leaf/EFLE	Stem/AR	Stem/EFLE
Cd	Min	0.03	0.03	0.03	0.01	0.23	71.2	0.03	0.01	0.01	0.06	0.00	0.06	0.02	0.15
	Max	2.18	1.25	1.16	0.51	12.4	3729	27.8	35.4	2.00	258	2.66	48.8	2.89	105
	Mean	0.40	0.55	0.29	0.16	1.94	583	9.91	5.30	0.35	16.4	0.34	4.67	0.68	12.4
	SE	0.13	0.07	0.08	0.03	0.58	173	2.03	1.96	0.12	11.1	0.13	2.17	0.14	5.27
Cu	Min	9.22	5.57	7.94	0.11	4.66	0.93	0.08	0.13	0.01	0.37	0.01	0.46	0.00	0.29
	Max	139	11.8	20.2	40.1	2363	473	0.75	1.50	7.52	9254	2.61	148	1.52	72.0
	Mean	27.0	7.71	12.9	3.41	242	43.3	0.40	0.70	1.49	504	0.90	50.0	0.53	29.2
	SE	5.68	0.30	0.71	1.99	128	23.1	0.04	0.08	0.34	398	0.16	8.88	0.09	4.85
Ni	Min	0.63	0.23	0.03	0.02	2.38	0.21	0.14	0.01	0.02	1.27	0.01	0.05	0.01	0.52
	Max	5.47	3.69	6.13	1.94	75.7	6.32	2.18	3.70	0.89	148	0.72	70.0	0.56	61.0
	Mean	2.14	1.48	2.18	0.39	16.1	1.42	0.83	1.18	0.25	23.6	0.20	9.78	0.14	10.6
	SE	0.26	0.18	0.35	0.09	3.59	0.31	0.11	0.21	0.05	8.70	0.04	2.97	0.03	3.25
Pb	Min	2.43	0.01	2.10	0.59	7.22	2.41	0.00	0.41	0.01	0.73	0.01	2.00	0.00	0.01
	Max	10.5	7.79	21.8	4.38	1004	323	1.14	4.02	0.37	86.1	1.35	14.6	0.38	5.10
	Mean	5.52	2.52	7.55	1.68	117	38.4	0.48	1.50	0.12	8.43	0.19	5.80	0.06	1.87
	SE	0.43	0.32	0.83	0.24	45.8	14.8	0.05	0.19	0.02	3.60	0.06	0.75	0.02	0.26
Zn	Min	50.7	26.9	18.7	0.05	11.0	0.17	0.47	0.25	0.02	0.00	0.01	0.74	0.01	0.76
	Max	300	246	160	130	3820	58.8	1.07	1.10	11.6	2931	3.33	481	7.99	674
	Mean	121	86.5	61.7	15.3	514	7.88	0.73	0.54	1.98	390	0.89	89.3	1.44	135
	SE	14.6	11.1	7.93	6.40	217	3.34	0.04	0.04	0.54	175	0.20	28.7	0.41	43.6

Note: min = minimum; max = maximum; SE = standard error.

**Table 4:** Correlation coefficients of metals between the plant parts (root, stem, and leaf) and their habitat topsoils (EFLE and total metal concentration (AR)). N = 23.

		EFLE	AR
Cd	Root	0.25 ^ns^	0.17 ^ns^
	Stem	0.09 ^ns^	0.03 ^ns^
	Leaf	0.04 ^ns^	0.03 ^ns^
Cu	Root	0.48 *	0.41 ^ns^
	Stem	0.54 *	0.48 *
	Leaf	0.30 ^ns^	0.26 ^ns^
Ni	Root	0.03 ^ns^	0.02 ^ns^
	Stem	0.33 ^ns^	0.48 *
	Leaf	0.40 ^ns^	0.36 ^ns^
Pb	Root	0.58 *	0.53 *
	Stem	0.29 ^ns^	0.05 ^ns^
	Leaf	0.42 *	0.28 ^ns^
Zn	Root	0.63 *	0.50 *
	Stem	0.56 *	0.44 *
	Leaf	0.69 *	0.60 *

Note: The correlation analysis was based on log_10_ transformed data of the metals. * = significant at *p* < 0.05; ^ns^ = not significant (*p* > 0.05).

**Table 5:** Plants under the medium of soils (except where indicated) employed for phytoremediation technologies through the process of phytoextraction, especially of Cd and Ni.

No.	Plants	Type	Contaminant (s)	Country	References
1	*Chromolaena odorata*	Invasive	Crude oil and Cd, Ni, Zn	South Africa	[71]
2	*Ipomoea carnea*	Invasive	Cd, Pb, Cu, Cr, Mn, and Ni	India	[72] *
3	*Amaranthus spinosus*	Invasive	Cu, Zn, Cr, Pb, and Cd	India	[73]
4	*Arundo donax*	Invasive	Improved pH, EC, OC, microbial counts, and soil enzyme activities and uptake Cd, Pb, Co, Ni, and Fe	Hungary	[74] **
5	*Typha latifolia*	Invasive	Zn, Mn, Cu, Pb, Cd, Cr, and Ni	India	[75]
6	*Typha latifolia*	Invasive	Al, As, Cd, Cr, Cu, Hg, Mn, Ni, Pb, and Zn	Italy	[76]
7	*Alternanthera philoxeroides*	Invasive	Cd	China	[77]
8	*Ambrosia artemisiifolia*	Invasive	As, Cd, Cr, Cu, Mn, Ni, Pb, V, and Zn	China	[78]
9	*Ageratum conyzoides*, *Bidens pilosa*, *Senecio scandens*, *Imperata cylindrical*, *Buddleja davidii*	Invasive	Cd, Pb, and Zn	China	[79]
10	*Chromolaena odorata*, *Bidens pilosa*, and *Praxelis clematidea*	Invasive	Cd	China	[68]
11	*Alyssum serpyllifolium* sp. *Lusitanicum*	Non-invasive	Ni	Portugal	[69]
12	*Celosia argentea*	Non-invasive	Cd	Field experiment	[70]
13	*Saccharum spontaneum* and *Saccharum munja*	Non-invasive	Zn, Pb, Cu, Ni, Cd, and As	Pot experiments	[80] ***
14	*Euphorbia helioscopia* and *Urtica dioica*	Non-invasive	As, Cd, Pb, Cu, and Zn	Bor (Serbia)	[40]
15	*Helianthus annuus*, *Brassica napus,* and *Chyrsopogon zizanioides*	Non-invasive	Cd	Greenhouse pot experiments	[81]
16	*Helianthus annuus*	Non-invasive	Cd	Experimental	[82]

Note: * medium was fly ash deposits; ** = medium was Bauxite-derived red mud; *** = included phytoextraction and phytostabilisation.

**Table 6:** Plants under the medium of soils employed for phytoremediation technologies with the process of phytostabilisation, especially of Cu, Pb, and Zn.

No.	Non-Invasive Plant (s)	Type	Metals	Country	References
1	*Phyla nodiflora*	Non-invasive	Cu and Zn	Field; North Florida, USA	[26]
2	*Gentiana pennelliana*	Non-invasive	Pb, Cu, and Zn	Field; North Florida, USA	[26]
3	*Festuca rubra*	Non-invasive	Pb and Mn	Field experiment	[93]
4	*Tamarix gallica*	Indigenous to Saudi Arabia and the Sinai Peninsula	Trace elements	pot experiment	[94]
5	*Lolium perenne*,	Native to Europe, Asia, and northern Africa	Cu, Pb, Mn, and Zn	highway soil in southwest British Columbia, Canada	[95]
6	*Loudetia simplex*	Native to Southern Africa and Madagascar	Cu	South D. R. Congo	[96]
7	*Tamarix africana*	Non-invasive	As, Cd, Cr, Cu, Pb, and Zn	Coina River	[97] *
8	*Piptatherum miliaceum*	Native to Eurasia	Trace elements	Sierra Minera of La Unión-Cartagena (SE Spain)	[98]
9	*Microchloa altera*	Non-invasive	Heavy metals	Democratic Republic of the Congo (DRC)	[99]
10	*Silybum marianum*, *Piptatherum miliaceum*, *Nicotiana glauca* and *Helianthus annuus*	Non-invasive	Trace metals	Pot experiment	[100]
11	*Spartina alterniflora*	Invasive	Cu, Zn, Pb, and Cr	China	[101] **
12	*Brassica campestris*	Non-invasive	Cd, Cu, Ni, Pb, and Zn	Botanical Garden of Komarov Botanical Institute, Russia	[91] ***
13	*Saccharum spontaneum* and *Saccharum munja*	Non-invasive	Zn, Pb, Cu, Ni, Cd, and As	Pot experiments	[80] ***
14	*Ulmus glabra*	Non-invasive	As, Cd, Cr, Cu, Ni, Pb, and Zn	Sava River	[92]

Note: * = saline-contaminated soils; ** = sediment; *** = Potentials as phytoextraction and phytostabilisation.

## Data Availability

Not applicable.
